# Reduced expression of erythropoietin-producing hepatocyte B6 receptor tyrosine kinase in prostate cancer

**DOI:** 10.3892/ol.2015.2925

**Published:** 2015-02-03

**Authors:** ELNISR RASHED MOHAMED, MASANORI NOGUCHI, AHMED ROSHDI HAMED, MOHAMED ZAKI ELDAHSHOURY, AHMED RASHED HAMMADY, ESAM ELDEN SALEM, KYOGO ITOH

**Affiliations:** 1 Department of Urology, Sohag University Hospital, Sohag 82425, Egypt; 2 Department of Pathology, Sohag University Hospital, Sohag 82425, Egypt; 3 Division of Clinical Research, Research Center for Innovative Cancer Therapy, Kurume University School of Medicine, Kurume, Fukuoka 830-0011, Japan; 4 Cancer Vaccine Center, Kurume University School of Medicine, Kurume, Fukuoka 830-0011, Japan

**Keywords:** erythropoietin-producing hepatocyte B6 expression, prostate cancer, cancer volume, receptor tyrosine kinase, biochemical progression-free survival

## Abstract

Loss of erythropoietin-producing hepatocyte (Eph) B6 gene expression is associated with poor prognosis in neuroblastoma, melanoma and other tumors. The present study evaluated the expression of EphB6 receptor tyrosine kinase in normal and prostate cancer tissue using immunohistochemistry. The association between EphB6 expression, clinicopathological findings, proliferating-cell nuclear antigen (PCNA; another prognostic marker) and progression of prostate cancer was analyzed. Tissue microarray samples of normal prostatic tissue and prostate cancer tissue from 46 patients treated with radical prostatectomy for prostate cancer were included in this study. Polyclonal anti-EphB6 and monoclonal anti-PCNA antibodies were used to assess EphB6 and PCNA expression by immunohistochemistry. EphB6 was expressed in normal and prostate cancer tissue; however, its expression was significantly reduced in prostate cancer tissue compared with normal prostatic tissue (P<0.0001), in high volume (≥4 cm^3^) cancer compared with low volume (<4 cm^3^; P=0.015), and in pT3 stage compared with pT2 stage of the disease (P=0.0007). No correlation was observed between the expression of EphB6 and PCNA. Short biochemical progression-free survival was associated with low EphB6 protein expression (P=0.157). This study revealed that EphB6 may have a tumor suppressor effect in prostate cancer, at least during early stages of this disease. This provides new insight into the potential utility of EphB6 receptor as a diagnostic/prognostic marker for prostate cancer.

## Introduction

The erythropoietin-producing hepatocyte (Eph) family of receptors is the largest family of receptor tyrosine kinases (RTKs) in humans (1). This family comprises 14 members associated with eight ephrin ligands. These receptors and ligands are divided into A and B classes based on their sequence homology and their affinity for their corresponding receptor/ligand (2–6). Many Eph receptors and ephrin ligands are known to be involved in the development or progression of malignant tumors: Upregulation of EphA2, A7, A10, and ephrinA2 and B3 is thought to be involved in tumorigenesis and/or invasiveness, while downregulation of EphA1, A3, A4, A8, B3, B4, B6, and ephrin A1 and B1 may be particularly important in tumor invasiveness (7). EphB6 is a clinically significant Eph receptor, as indicated by its loss in the most aggressive forms of melanoma and neuroblastoma (8–10). Loss of EphB6 is associated with angiogenesis and tumor vasculature in several types of human cancer (7,11,12). However, the reports with regard to the role of Eph RTK members, particularly EphB6, in prostate cancer, are insufficient.

In the present study, the expression of EphB6 receptor in normal and prostate cancer tissue, and the association between EphB6 expression, clinicopathological findings and progression of prostate cancer was investigated. Additionally, the potential association between the expression of EphB6 and proliferating-cell nuclear antigen (PCNA), an independent postoperative prognostic marker for prostate cancer patients (13), was assessed.

## Materials and methods

### Tissue samples

The protocol was approved by the ethics committee of Kurume University (Kurume, Japan). Between 2003 and 2005, 46 patients were enrolled in the study and underwent radical prostatectomy for prostate cancer at Kurume University Hospital (Kurume, Japan). Following a full explanation of the protocol, written informed consent for the use of tissue samples was obtained from all patients prior to enrollment. Patients with clinically localized prostate cancer who underwent radical retropubic prostatectomy were enrolled, however, patients treated with hormones, irradiation or transuretheral resection prior to surgery were excluded.

Prostatectomy specimens were evaluated using the following technique, with sectioning performed at 3 mm intervals. The grade of each tumor was determined according to the Gleason system of five grades (14). In each patient, the volume of the cancer was determined using a computer-assisted image analysis system (15). Seminal vesicle invasion and positive regional lymph nodes were recorded. A tissue microarray of the prostate was constructed as previously described (16). Briefly, one donor block, which included normal and tumor regions, was selected from 10–15 blocks of formalin-fixed, paraffin-embedded prostate tissue from each patient. Tissue cylinders, with a diameter of 2 mm, were subsequently punched from six regions of normal and cancer tissue in each donor block, using a tissue microprocessor instrument (KIN-type I, AZUMAYA, Tokyo, Japan), and inserted into a recipient paraffin block. Tissue blocks from 46 prostatectomy specimens were evaluated for the expression of EphB6 and PCNA using immunohistochemistry.

### Immunohistochemistry

Rabbit polyclonal antibody (EphB6 antibody, H-90; cat. no. sc-25461; Santa Cruz Biotechnology, Inc., Santa Cruz, CA, USA) at a dilution of 1:500 and mouse monoclonal (PCNA antibody, PC-10; cat. no. M0879; Dako Corporation, Glostrup, Denmark) IgG at a dilution of 1:200 were used to evaluate the expression of EphB6 and PCNA, respectively, by immunohistochemistry. Briefly, 5 μm thick sections of the selected paraffin blocks were de-paraffinized in xylene, rehydrated in graded alcohol, and incubated in 0.5% hydrogen peroxide/methanol for 20 min to block endogenous peroxidase activity. Antigen retrieval was conducted by boiling the sections in a microwave for 10 min using 10 mM citrate buffer (pH 6.0). The sections were subsequently incubated with anti-EphB6 or anti-PCNA antibody overnight at 4°C. Following this incubation, the sections were washed with 0.5% Tween-20/phosphate buffered saline (PBS) prior to incubation with the corresponding polyclonal peroxidase-labeled goat anti-rabbit and goat anti-mouse secondary antibodies (dilution 1:100; Histofine, Nichirei, Tokyo, Japan) for 60 min. The sections were subsequently washed with 0.5% Tween-20/PBS, and exposed to 3,3′-diaminobenzidine tetrahydrochloride solution (Dako, Carpinteria, USA) to yield an insoluble brown deposit. Finally, the sections were counterstained with hematoxylin, washed in running water, dehydrated in graded alcohol and conventionally mounted. Replacement of the primary antibodies with PBS was used as a negative control for the immunohistochemistry process.

### Scoring of immune reactions

The immunoreactivity of EphB6 and PCNA molecules was evaluated without prior knowledge of the clinicopathological findings. The staining intensity of EphB6 was scored as 0 (negative) when immunoreactivity was absent or present in <10% of cells, and as 1 (weak), 2 (moderate) and 3 (strong) when present in 10–20%, 20–50% and >50% of cells, respectively. The PCNA labeling index (LI) was determined by counting 1000 tumor cells at ×400 magnification in 10 randomly selected microscopic fields. Brown, granular nuclear staining was considered positive staining. The PCNA LI was calculated as the percentage of tumor cells with positive nuclear staining for PCNA (13).

### Statistical analysis

SPSS version 19.0 for Windows (IBM SPSS, Armonk, NY, USA) was used for data analysis. P<0.05 was considered to indicate a statistically significant difference. The change in EphB6 expression in prostate cancer compared with that of corresponding normal tissue was assessed using Wilcoxon’s signed rank test. The frequency of a categorical observation was compared between different groups using the χ^2^ and Fisher’s exact tests, and the correlation between expression status of EphB6 and other continuous variables was evaluated by Spearman’s ρ test. Mann-Whitney U and Kruskal-Wallis tests were used to compare the mean rank of continuous variables between different clinical groups and Kaplan-Meier survival analysis was used to compare the duration of prostate-specific antigen (PSA)-free survival between the different groups.

## Results

### Patient characteristics

The demographic characteristics of the investigated patients and pathological features of the tumors are summarized in Table I. Patients were aged from 49–78 years (mean, 65.8 years; median, 66 years). Serum PSA was elevated in all patients, with a minimum value of 4.4 ng/ml and a maximum of 33.9 ng/ml (normal range, <4 ng/ml). The majority of tumors (56.5%) were of Gleason score 7. Cancer volume in prostatic specimens ranged from 0.2–15.2 cm^3^ (median, 3.7 cm^3^). Pathological T stages were pT2a in 16, pT2b in seven, T2c in nine, pT3a in 10 and pT3b in four patients. Nodal or distant metastasis was not detected in any of the patients.

### Expression of EphB6

**Figure 1:**
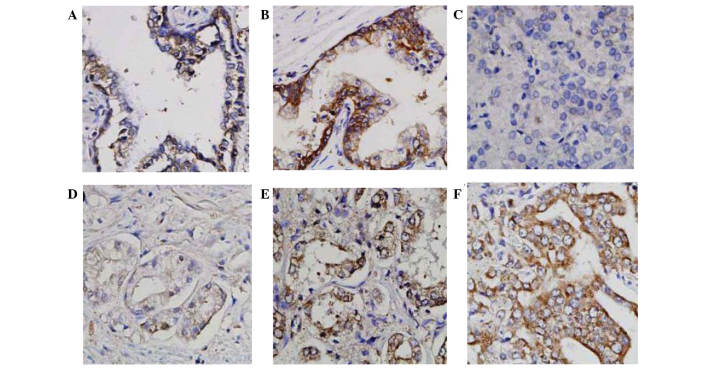
Expression of EphB6 in normal and prostatic cancer tissue. Normal prostatic acini expressed EphB6 at either (A) moderate or (B) strong levels, while prostatic cancer tissue showed (C) negative, (D) weak, (E) moderate or (F) strong expression levels of EphB6. Magnification, ×400. Eph, erythropoietin-producing hepatocyte.

The expression of EphB6 was evaluated in normal and prostate cancer tissues from each patient. In normal prostatic tissues, EphB6 expression was observed in 100% of investigated samples. EphB6 protein had a homogeneous cytoplasmic and membranous distribution, and the immunoreactivity was either moderate or strong (38% and 62% of samples, respectively; Fig. 1A and B). In prostate cancer tissue, EphB6 expression was detected in the majority of cases (97.8%). The distribution was also membranous and cytoplasmic, and the expression level was negative or weak in a high proportion of cases (26 cases, 56.5%) and moderate or strong in 17 (37%) and three (6.5%) cases, respectively (Fig. 1C–F). Compared with corresponding normal tissue within the same patient, prostate cancer cells showed a significantly decreased expression level of EphB6 in 26 cases and retained a similar expression level in the remaining cases (Wilcoxon signed rank test, P<0.0001).

### Expression of PCNA

**Figure 2:**
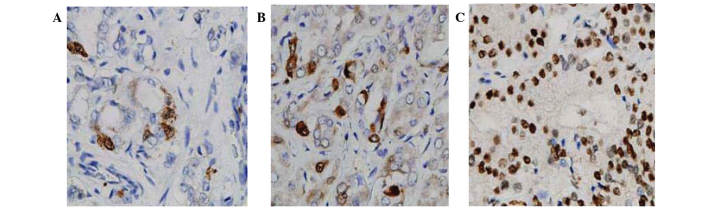
Expression of PCNA in prostate cancer tissue. Nuclear expression of PCNA with labeling index of (A) 18%, (B) 35% and (C) 85%. Magnification, ×400. PCNA, proliferating-cell nuclear antigen.

The expression of PCNA, which is predominantly nuclear, was detected in all investigated patients. The minimum LI of PCNA was 1% and the maximum was 98%, with a median value of 22%. Representative PCNA expressions in prostate cancer are shown in Fig. 2. No significant association between the expression status of PCNA and EphB6 was observed (Spearman’s ρ=0.193, P=0.173).

### Association between tumor characteristics and EphB6 or PCNA expression

**Figure 3:**
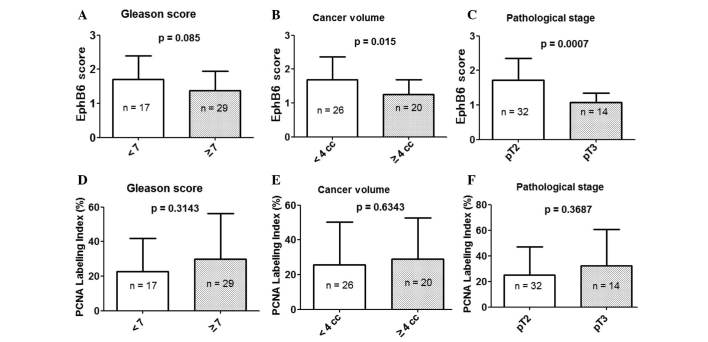
Association between tumor characteristics and EphB6 or PCNA expression. Association between EphB6 staining score and (A) Gleason score, (B) cancer volume and (C) pathological stage. Association between PCNA labeling index and (D) Gleason score, (E) cancer volume and (F) pathological stage. Eph, erythropoietin-producing hepatocyte; PCNA, proliferating-cell nuclear antigen; cc, cm^3^.

The association between tumor characteristics (including Gleason score, cancer volume and pathological stage) and EphB6 or PCNA expression were evaluated (Fig. 3). The results revealed that low expression of EphB6 was significantly associated with a high volume (≥4 cm^3^) of cancer (P=0.015) and advanced pathological stage (pT3) (P=0.0007). However, none of the tumor characteristics were associated with PCNA expression.

### EphB6 expression and biochemical progression-free survival

**Figure 4:**
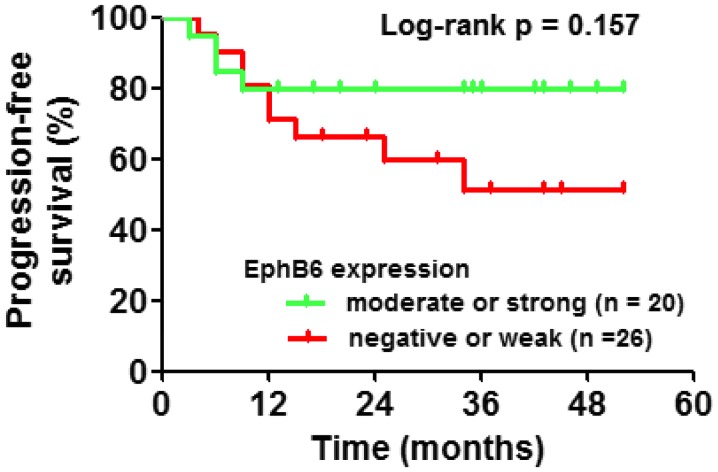
Kaplan-Meier plot showing time to biochemical progression-free survival of prostate cancer patients in different EphB6 expression groups. Eph, erythropoietin-producing hepatocyte.

The effect of EphB6 expression on biochemical progression-free (PSA-free) survival in prostate cancer patients was evaluated (Fig. 4). The minimum follow-up duration of the investigated patients was 12 months and the maximum was 120 months (median, 47.5 months). According to the Kaplan-Meier analysis, biochemical progression-free survival was reduced in patients with negative or weak expression of EphB6, compared with that of patients with mild or strong expression (hazard ratio, 2.227; 95% CI, 0.7353–6.745; log-rank, P=0.157).

## Discussion

An understanding of tumor pathogenesis and the identification of prognostic and diagnostic molecules are crucial in the management of prostate cancer. A number of studies have reported that the Eph RTK family of receptors and their ephrin ligands enhance tumor growth, invasion, metastasis and neovascularization (17,18). Previous studies observed that expression of EphB6 was diminished or lost in the most aggressive forms of melanoma and neuroblastoma (8–10). Furthermore, forced expression of EphB6 in neuroblastoma cells may decrease their tumorigenicity in mouse xenograft models (9). In the current study, normal prostatic tissue exhibited homogeneous moderate or strong expression of EphB6 in 15 (33%) and 31 (67%) of the investigated cases, respectively. In addition, significantly reduced EphB6 expression was observed within adjacent prostate cancer tissue in a considerable proportion of cases (Wilcoxon’s signed rank test, P<0.0001). This is consistent with previous semi-quantitative RT-PCR studies on prostate cancer cell lines, which showed downregulation of EphB6 mRNA in a cell line derived from primary prostate cancer tissue, compared with that in a cell line derived from normal prostatic tissue from the same patient (19). These findings support the hypothesis that EphB6 is a tumor suppressor molecule in prostate cancer and that its expression is correlated with favorable tumor prognosis. Additionally, data from the current study suggested that EphB6 expression is gradually and significantly reduced during the progression of prostate cancer from a low volume to a high volume, or from pT2 stage to pT3. Furthermore, no association was observed between EphB6 expression and the expression of PCNA. Within the limits of the investigated cases, the results indicate that EphB6 RTK has no proliferation-stimulating effect in prostate cancer.

In apparent contradiction with the hypothesized tumor suppressor effect of the EphB6 molecule in prostate cancer, Fox *et al* (19) reported that the invasive and metastasizing prostate cancer cell lines DU145, PC-3 and PC-3ML exhibited upregulation of EphB6 mRNA compared with that of cell lines derived from primary prostate cancer or normal tissue. These controversial observations are not fully understood; however, they may be associated with a change in the subcellular localization of the EphB6 molecule. Additionally, the regulation of EphB6 expression by promoter methylation may be associated with altered expression in aggressive prostate cancer cell lines (19).

In conclusion, although several members of the Eph family are associated with the progression of cancer, the results of the present study indicated that EphB6 may have a tumor suppressor effect in prostate cancer, at least during early stages of this disease. This provides new insight for the use of EphB6 RTK as a potential diagnostic/prognostic marker for prostate cancer. However, a significant limitation of the current study is the inclusion of only early stages of prostate cancer. Further studies of EphB6 protein and mRNA expression in later stage and metastatic prostate cancer tissue are required in order to fully evaluate the role of EphB6 in this disease, and to address the aforementioned conflicting results from other studies.

## Figures and Tables

**Table I:** The demographic characteristics of patients and tumors.

Clinicopathological factors	
Patients, n	46
Age, years	
Mean (SD)	65.8 (6.2)
Median (range)	66 (49–78)
Serum PSA level, ng/ml	
Mean (SD)	13.2 (7.5)
Median (range)	11.4 (4.4–33.9)
Prostatic weight, gm	
Mean (SD)	27.5 (10.0)
Median (range)	26.2 (11.8–60.8)
Gleason score, n (%)	
6	17 (37)
7	26 (56.5)
8	1 (2)
9	2 (4.5)
Pathological T stage, n (%)	
pT2a	16 (35)
pT2b	7 (15)
pT2c	9 (20)
pT3a	10 (21.5)
pT3b	4 (8.5)
Cancer volume, cm^3^	
Mean (SD)	4.8 (3.9)
Median (range)	3.7 (0.2–15.2)

SD, standard deviation; PSA, prostate-specific antigen.

## Abbreviations

Eph: erythropoietin-producing hepatocyte
LI: labeling index
PCNA: proliferating-cell nuclear antigen
PSA: prostate-specific antigen
RTK: receptor tyrosine kinase
TNA: tissue microarray
